# Folate Receptor Alpha in Advanced Epithelial Ovarian Cancer: Diagnostic Role and Therapeutic Implications of a Clinically Validated Biomarker

**DOI:** 10.3390/ijms26115222

**Published:** 2025-05-29

**Authors:** Gian Franco Zannoni, Angela Santoro, Antonio d’Amati, Nicoletta D’Alessandris, Giulia Scaglione, Belen Padial Urtueta, Michele Valente, Nadine Narducci, Francesca Addante, Saveria Spadola, Emma Bragantini, Giuseppe Angelico

**Affiliations:** 1 Pathology Unit, Department of Woman and Child’s Health and Public Health Sciences, Fondazione Policlinico Universitario Agostino Gemelli IRCCS, 00168 Rome, Italy; angela.santoro@policlinicogemelli.it (A.S.); antonio.damati@uniba.it (A.d.); nicoletta.dalessandris@policlinicogemelli.it (N.D.); giulia.scaglione@policlinicogemelli.it (G.S.); belen.padialurtueta@guest.policlinicogemelli.it (B.P.U.); dr.valente.m@gmail.com (M.V.); nadine.narducci@libero.it (N.N.); francesca.addante1@gmail.com (F.A.); 2 Pathology Institute, Catholic University of Sacred Heart, 00168 Rome, Italy; 3 Department of Medicine and Surgery, Kore University of Enna, 94100 Enna, Italy; saveriaspadola@hotmail.it (S.S.); giuangel86@hotmail.it (G.A.); 4 Pathology Unit, Cannizzaro Hospital, 95126 Catania, Italy; 5 Ospedale San Maurizio, 39100 Bolzano, Italy; emma.bragantini@sabes.it

**Keywords:** folate receptor alpha (FRα), immunohistochemistry, ovarian cancer, mirvetuximab soravtansine, platinum-resistant epithelial ovarian cancer

## Abstract

Folate receptor alpha (FRα), a glycosylphosphatidylinositol-anchored glycoprotein encoded by the *FOLR1* gene, plays a crucial role in folate transport during cell growth and development. While minimally expressed in most normal adult tissues, FRα is frequently overexpressed in several epithelial malignancies, particularly in high-grade serous ovarian carcinoma. An immunohistochemical (IHC) evaluation of FRα expression using the VENTANA FOLR1 (FOLR1-2.1) RxDx Assay is now approved as a companion diagnostic for selecting patients eligible for mirvetuximab soravtansine, an FRα-targeted antibody–drug conjugate. Clinical trials such as SORAYA and MIRASOL have demonstrated significant clinical benefit in platinum-resistant epithelial ovarian cancer patients with high FRα expression (≥75% of tumor cells with moderate to strong membrane staining). This review summarizes the biological significance of FRα in ovarian cancer progression, its predictive value for targeted therapy, and the technical aspects of IHC assessment, including scoring interpretation and pre-analytical variables. We also discuss heterogeneity in FRα expression across histological subtypes and tumor sites, as well as the impact of archival versus fresh tissue. Understanding FRα expression patterns across histologic subtypes and tissue samples is critical for optimizing clinical decision-making and expanding the role of FRα-targeted therapies in gynecologic oncology.

## 1. Introduction

Folate is a water-soluble vitamin essential for DNA and RNA synthesis in normal cell growth and development, being first isolated in 1972 from cow’s milk and identified as a cancer-associated antigen in ovarian cancer in 1991 [1]. Folate receptor alpha (FRα) is a 38–40 kDA glycosylphosphatidylinositol (GPI)-anchored cell-surface glycoprotein encoded by the *FOLR1* gene, characterized by high affinity for folate, its derivatives, and folate analogs [2]. It is defined as a low-throughput transporter, transferring folate through endocytosis in selected tissues and providing folate for one-carbon metabolism, cell growth, development, and proliferation [2,3]. In normal tissues, FRα can be localized to the luminal surface of certain epithelial cells, where it is inaccessible to the circulation, whereas in tumors, it is fully accessible via the circulation [2,3,4].

FRα expression is limited in most (not all) normal adult tissues, and folate intake cells are believed to be largely provided by the reduced folate carrier, characterized by a low affinity for folate, being a high-throughput transporter [2,3,4]. On the other hand, FRα is frequently overexpressed during physiological processes characterized by rapid cell growth, such as pregnancy and embryogenesis. It is also overexpressed in various epithelial malignancies, with reported frequencies ranging from 35 to 70% in breast cancer, 15 to 75% in lung cancer, 20 to 50% in endometrial cancer, and 75 to 90% in ovarian cancer, particularly in high-grade serous ovarian carcinoma, where elevated expression is observed in approximately 60–85% of cases. These percentages are derived from the literature and reflect variability in detection methods, sample sizes, and expression thresholds [2,3,4].

FRα levels are positively associated with tumor stage and grade, which suggests that FRα might confer a tumoral growth advantage by modulating folate uptake or by generating regulatory signals [5].

In ovarian cancer, FRα contributes to tumor growth through both its role in folate transport and through additional folate-independent mechanisms [6]. By enhancing folate uptake, FRα supports DNA and RNA synthesis, which is essential for the rapid cell division characteristic of malignant tumors [6]. Beyond this metabolic function, FRα also activates key oncogenic signaling pathways such as JAK/STAT3 and MAPK/ERK, which promote cell proliferation, survival, and resistance to apoptosis [6]. It further influences the expression of genes associated with stemness, epithelial-to-mesenchymal transition, and tumor progression, contributing to a more aggressive cancer phenotype [6]. There is also evidence that FRα may modulate the tumor microenvironment and immune response, helping cancer cells evade immune detection [6].

The above-mentioned features suggest the role of FRα as a valuable biomarker and a promising target for precision medicine in ovarian cancer.

The aim of this review is to provide an overview of folate receptor alpha (FRα) expression in ovarian cancer, with a particular focus on its immunohistochemical evaluation using the Ventana FOLR1 RxDx Assay. We discuss the biological role of FRα in tumor development and progression, its prevalence across different histological subtypes of ovarian carcinoma, and its potential as a predictive biomarker for targeted therapies. The review also addresses the interpretation criteria, scoring systems, and technical considerations involved in FRα immunostaining, including the importance of internal control tissue for assay validation.

## 2. Mirvetuximab Soravtansine-Gynx Clinical Trials

Clinically, FRα has gained considerable interest as a biomarker for selecting patients eligible for targeted therapies, particularly antibody–drug conjugates (ADCs) such as mirvetuximab soravtansine-gynx (ELAHERE^®^) [7]. The efficacy of this ADC against epithelial ovarian cancer (EOC) has been investigated in several clinical trials as monotherapy or in combination with other drugs [7,8,9,10,11,12,13]. The therapeutic benefit of these agents appears closely related to the degree of FRα expression in tumor tissue, highlighting the importance of accurate and reproducible immunohistochemical (IHC) assessment [7,8,9,10,11,12,13]. Mirvetuximab is an ADC consisting of a humanized monoclonal antibody targeting folate receptor alpha (FRα), conjugated via a cleavable linker to the maytansinoid DM4, a potent tubulin inhibitor [7,8,9,10,11,12,13]. The agent is designed to selectively bind FRα-expressing tumor cells and internalize via receptor-mediated endocytosis. Upon intracellular degradation of the ADC, the cytotoxic drug is released, thereby inducing apoptosis while minimizing off-target [7,8,9,10,11,12,13]. The clinical activity of mirvetuximab has been evaluated across several trials in patients with platinum-resistant epithelial ovarian cancer, where FRα expression represented a crucial biomarker for therapeutic efficacy [7,8,9,10,11,12,13]. All trials used the VENTANA FOLR1 (FOLR1-2.1) RxDx Assay to determine patient FRα expression [14]. The initial Phase III trial, FORWARD I, enrolled patients with ≥50% of tumor cells exhibiting any membrane staining for FRα. While the study failed to meet its primary endpoint of improved progression-free survival (PFS) in the intent-to-treat population, exploratory analysis suggested a clinical benefit in patients with high FRα expression levels [15].

Building on these findings, the single-arm Phase II SORAYA trial focused on a biomarker-enriched population, including only patients with platinum-resistant, advanced high-grade epithelial ovarian, primary peritoneal, or fallopian tube cancers, whose tumors demonstrated ≥75% of tumor cells with moderate to strong FRα membrane staining, as determined by the Ventana FOLR1 assay [16]. In this cohort, mirvetuximab achieved an objective response rate (ORR) of 32.4%, with a median duration of response of 6.9 months and a manageable safety profile. These promising results supported the FDA’s accelerated approval of mirvetuximab in November 2022 [16].

The confirmatory randomized Phase III MIRASOL trial further validated the clinical utility of mirvetuximab in the population of platinum-resistant, advanced high-grade epithelial ovarian, peritoneal, or fallopian tube cancers, with FRα-high expression (≥75% of tumor cells with 2+/3+ staining) [13]. Compared to the investigator’s choice chemotherapy, mirvetuximab significantly improved median PFS (5.62 vs. 3.98 months) and overall survival (16.46 vs. 12.75 months), with an ORR of 42.3% versus 15.9% in the control arm. This represented a 33% reduction in the risk of death and established mirvetuximab as the first agent to demonstrate an overall survival advantage in this setting. Based on these data, both the FDA and EMA granted full approval to mirvetuximab in 2024 [13].

Looking forward, several therapeutic avenues are under exploration. These include evaluating mirvetuximab soravtansine in earlier treatment lines for high-grade serous ovarian cancer (HGSOC) and in combination strategies with anti-angiogenic agents [17]. The rationale for combining this ADC with bevacizumab is supported by their non-overlapping toxicity profiles and potential synergistic effects, as previously observed in the AURELIA trial [17]. The FORWARD II study confirmed that combining mirvetuximab with bevacizumab in platinum-resistant patients is feasible and active, paving the way for future synergistic approaches [18].

The GLORIOSA trial (NCT05445778) is an ongoing randomized Phase III study evaluating mirvetuximab plus bevacizumab as maintenance therapy in patients with platinum-sensitive ovarian cancer and high FRα expression, aiming to prolong progression-free survival compared to bevacizumab alone [19]. In parallel, the PICCOLO trial is assessing mirvetuximab monotherapy in platinum-sensitive patients with high FRα levels after multiple prior platinum-based treatments, showing a promising ORR of 47% in preliminary data [12].

## 3. Ventana FOLR1 (FOLR1 2.1) RxDx Assay

**Figure 1:**
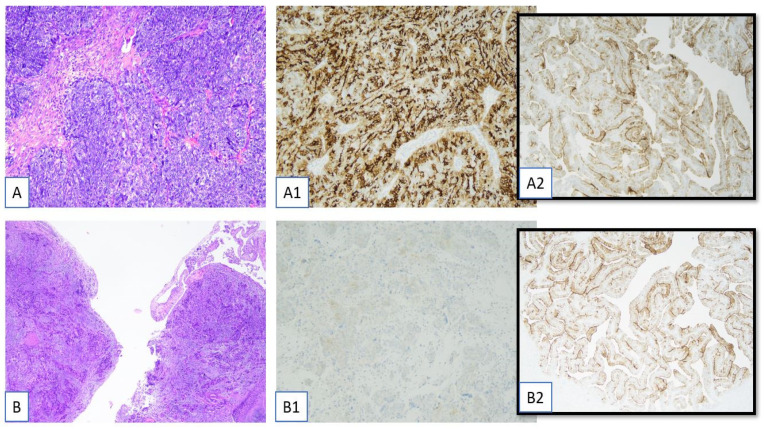
Immunohistochemical determination of folate receptor alpha (Ventana FOLR1 RxDx Assay-FOLR1-2.1 antibody on Benckmark Ultra platform, Roche Diagnostics International AG, Rotkreuz, Switzerland). (**A**) High-grade serous carcinoma of the salpinx (H&E, 10×). (**A1**) FOLR1 membrane positivity (score 2+/3+) in 90% of tumor cells (Ventana FOLR1 RxDx Assay-FOLR1-2.1 antibody on Benckmark Ultra platform, LSAB-HRP, 10×). (**A2**) Adequate control tubal tissue (4×). (**B**) Peritoneal metastasis of ovarian High-Grade Serous Carcinoma (H&E, 4×). (**B1**) FOLR1 membrane positivity (score 2+/3+) in <5% of tumor cells (Ventana FOLR1 RxDx Assay-FOLR1-2.1 antibody on Benckmark Ultra platform, LSAB-HRP, 10×). (**B2**) Adequate control tubal tissue (4×).

The Ventana FOLR1 Assay showed robust accuracy and consistency in detecting FRα expression and selecting patients eligible for mirvetuximab treatment [20]. This immunohistochemical test utilizes a mouse monoclonal anti-FOLR1 antibody (clone FOLR1-2.1) to detect folate receptor alpha (FOLR1) protein expression in formalin-fixed, paraffin-embedded (FFPE) tissue specimens from epithelial ovarian, fallopian tube, or primary peritoneal cancers [14]. The assay is designed for use with the OptiView DAB IHC Detection Kit on the BenchMark IHC/ISH automated staining platform [14]. FOLR1 expression clinical cut-off is ≥75% viable tumor cells (TC) with membrane staining at moderate (2+) and/or strong (3+) intensity levels [14,20,21,22] (Figure 1).

The assay is indicated as the first and only analytically and clinically validated, FDA and CE IVD-approved IHC companion diagnostic for determining FRα protein expression to aid in identifying patients with epithelial ovarian, fallopian tube, or primary peritoneal cancer who may be eligible for treatment with ELAHERE (mirvetuximab soravtansine) [14,20,21,22,23].

Despite the limited number of studies on this topic, current recommendations based on available scientific evidence emphasize that results from the VENTANA FOLR1 (FOLR1-2.1) RxDx Assay should be interpreted by a qualified pathologist, in conjunction with histological evaluation, relevant clinical information, and appropriate controls [14,20,21,22,23].

### 3.1. Pre-Analytical Considerations for FOLR1 Immunohistochemistry

Validated specimens for FOLR1 immunohistochemical assay include formalin-fixed, paraffin-embedded (FFPE) tissue samples from primary ovarian, fallopian tube, and peritoneal cancers [14,20,21,22,23]. These may consist of either archival or recent specimens obtained through resections, excisions, or biopsies and can include both primary and metastatic sites [14,20,21,22,23]. Proper specimen handling is critical to ensure accurate and reproducible results. Tissues should be fixed in 10% neutral buffered formalin for 12 to 72 h; a minimum of 6 h is required for small biopsies [14,20,21,22,23].

Tissue sections must be cut at a thickness of 4 μm and mounted on positively charged slides, with cut slide stability limited to 45 days. Specimens such as cytology samples and decalcified metastatic bone lesions are not validated for use with this assay [14,20,21,22,23].

To ensure assay reliability and performance, the use of a System Level Control (SLC) is essential. The SLC serves as a quality checkpoint, verifying that staining instruments, automated platforms, and manual processes are functioning properly [14,20,21,22]. It also confirms that primary and secondary antibodies, along with detection reagents, are performing as expected while also helping to detect batch-to-batch variability or shifts in assay performance over time [14,20,21,22,23]. For this purpose, tissue with staining intensity near the clinically relevant threshold is used; typically, normal fallopian tube epithelium demonstrates predominantly moderate (2+) circumferential FOLR1 membrane staining without staining in the underlying stroma [14,20,21,22,23]. While strong (3+) apical staining may be observed, it is not considered in scoring [14,20,21,22,23]. An unacceptable SLC result is defined by either absence of staining, predominance of weak (1+) or excessively strong (3+) circumferential staining, or nonspecific background staining that interferes with interpretation [14,20,21,22,23]. It is important to note that not all fallopian tube tissues will stain adequately; when this occurs, it reflects tissue inadequacy rather than assay failure. Each laboratory should identify and qualify fallopian tube tissue capable of serving as a consistent internal control, thereby ensuring stable assay performance over time [14,20,21,22,23]. Table 1 provides an overview of the technical characteristics of the VENTANA FOLR1 (FOLR1-2.1) RxDx Assay, including validated specimen types, scoring cut-offs, and platform specifications used in clinical diagnostics.

### 3.2. Pathological Evaluation of FRα: Scoring Criteria

Epithelial ovarian cancer (EOC) specimens demonstrate a range of folate receptor alpha (FRα) immunohistochemical staining intensities, categorized as weak, moderate, or strong [14,20,21,22,23]. A score of 0 is assigned when no detectable signal is present, although minimal pale grey cytoplasmic or membranous discoloration may occasionally be observed in negative cases. A score of 1+ corresponds to a faint membranous signal—typically light brown or golden in color—that may be partial or circumferential. A score of 2+ reflects moderate membranous staining, while a score of 3+ indicates strong membranous staining characterized by a thick, dark brown to black hue; both 2+ and 3+ signals may be either partial or circumferential.

**Figure 2:**
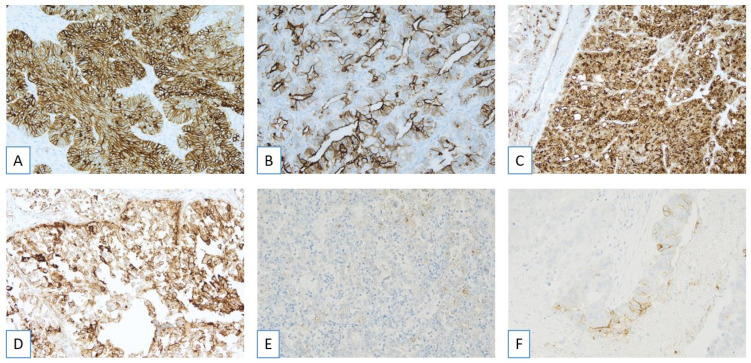
Immunohistochemical determination of folate receptor 1 (alpha receptor) status (Ventana FOLR1 RxDx Assay-FOLR1-2.1 antibody on Benckmark Ultra platform): accepted (**A**–**C**) and unaccepted patterns (**D**–**F**). (**A**) FOLR1 circumferential membrane positivity (score 2+/3+) in tumor cells (FOLR1, LSAB-HRP, 20×). (**B**) FOLR1 apical pattern positivity (score 2+/3+) in tumor cells (FOLR1, LSAB-HRP, 20×). (**C**) FOLR1 dot pattern positivity (score 2+/3+) in tumor cells (FOLR1, LSAB-HRP, 10×). (**D**) FOLR1 cytoplasmic positivity (score 2+/3+) in tumor cells (FOLR1, LSAB-HRP, 20×). (**E**) FOLR1 membrane positivity (score 1+) in tumor cells (FOLR1, LSAB-HRP, 20×). (**F**) FOLR1 membrane positivity (score 2+) in tumor cells (FOLR1, LSAB-HRP, 20×).

For scoring purposes, only membranous staining of moderate (2+) or strong (3+) intensity is considered, regardless of whether the staining is complete or incomplete [14,20,21,22,23]. Accepted staining patterns include circumferential, apical, and dot-like localization (Figure 2). In tumor cells exhibiting variable staining intensities, the highest intensity observed within each cell is recorded [14,20,21,22,23]. Cytoplasmic staining is excluded from the evaluation, and care must be taken to avoid over-scoring when both membranous and cytoplasmic staining are present by focusing exclusively on the membranous component [14,20,21,22,23].

Cells affected by necrosis, crush artifacts, or thermal damage are excluded from evaluation. Similarly, membranous staining of 0 or 1+ intensity, as well as purely cytoplasmic staining, are not included in the final assessment [14,20,21,22,23].

Based on these criteria, tumors are classified as FRα-positive if ≥75% of viable tumor cells demonstrate moderate (2+) and/or strong (3+) membranous staining, including apical or dot-like patterns [14,20,21,22,23]. Tumors are considered FRα-negative if fewer than 75% of viable tumor cells meet these criteria. Cases are designated as non-evaluable or indeterminate when technical artifacts preclude reliable interpretation [14,20,21,22,23]. The criteria for scoring FRα expression by immunohistochemistry are summarized in Table 2.

### 3.3. Pathological Evaluation of FRα: Complex Staining Patterns and Borderline Cases

Although infrequent, certain cases may exhibit staining features that complicate interpretation. Dot-like and apical staining patterns are considered positive, provided they are associated with membranous localization. In cases of apical staining, the entire cell is scored as positive [14,20,21,22,23]. Dot-like staining, typically observed as luminal punctate signals, warrants consideration of the entire affected gland as positive [14,20,21,22,23]. In tumors with heterogeneous staining, careful evaluation of both stained and unstained regions is essential, with the highest intensity within each cell recorded for scoring purposes [14,20,21,22,23].

Borderline cases, defined as those within ±10% of the 75% positivity threshold (i.e., 65–85% of tumor cells demonstrating 2+ or 3+ staining), present significant interpretive challenges [14,20,21,22,23]. In such instances, assessment at low magnification to encompass the full tumor area may aid in estimating the percentage of positive cells. These cases should be reviewed by additional pathologists, and a consensus approach or majority decision is recommended [14,20,21,22,23]. When feasible, retesting using an alternative formalin-fixed, paraffin-embedded tissue block may enhance diagnostic reliability.

### 3.4. Immunohistochemical Report of the Ventana FOLR1 RxDx Assay

Accurate immunohistochemical evaluation of folate receptor alpha (FOLR1) expression is critical for identifying patients with ovarian carcinoma who may benefit from targeted therapy with Mirvetuximab Soravtansine. To ensure reproducibility and diagnostic accuracy, pathology reports must comprehensively address specific technical, morphological, and interpretive parameters [14,20,21,22,23]. The following key elements should be systematically included in any formal immunohistochemical report using this assay [14,20,21,22,23]:

- **Assay methodology:**
  The report should state that the evaluation was performed using the Ventana FOLR1 RxDx Assay, which employs the FOLR1-2.1 monoclonal antibody on the Benchmark Ultra IHC platform. This ensures standardization and clinical validation of the assay for determining FOLR1 status in ovarian cancer.
- **Tumor histotype:**
  The histopathological classification of the tumor must be clearly specified. Most commonly, the assay is applied to high-grade serous carcinoma (HGSC), but other histological subtypes should be explicitly mentioned when applicable.
- **Anatomic location of the tissue sample:**
  The report should identify whether the analyzed tissue is from a primary site (ovary, salpinx, or peritoneum) or a metastatic lesion, as this may influence the interpretation and clinical implications.
- **Internal control tissue evaluation:**
  The presence of normal fallopian tube epithelium on the same slide is essential for internal quality control. The report must document whether control tissue is present and morphologically adequate (i.e., well preserved and with an expected staining pattern), which confirms the technical validity of the assay.
- **Assessment of tumor cell adequacy:**
  It is critical to confirm that the tissue section contains a minimum of 100 viable tumor cells. If this threshold is not met, the report should specify the reason for inadequacy (e.g., artifacts from freezing or fixation, necrosis, or absence of invasive tumor tissue), and the case may be considered non-evaluable.
- **Scoring procedure and interobserver agreement:**
  The report should state whether shared scoring was performed among multiple observers (e.g., consensus reading by two or three pathologists) or if a single observer evaluated the case. Shared scoring improves reproducibility and reduces interobserver variability in borderline or complex cases.
- **Quantitative scoring results:**
  For each participating observer (e.g., Operators I, II, III), the report should indicate the percentage of tumor cells showing membranous staining with moderate (2+) or strong (3+) intensity. This data supports both the final interpretation and the transparency of evaluation.
- **Use of additional slides for reevaluation:**
  If the initial slide was suboptimal or yielded an equivocal result, the report should document whether reevaluation on other included tissue sections was performed and whether it affected the final interpretation.
- **Final interpretive classification:**
  Based on established criteria, the tumor should be classified as FOLR1-positive, FOLR1-negative, or indeterminate. This classification must integrate staining intensity, percentage of positive tumor cells, and internal control status.
- **Therapeutic implications:**
  The report should conclude with a clinical interpretation indicating whether the patient is eligible, not eligible, or indeterminate for treatment with mirvetuximab soravtansine, an antibody–drug conjugate approved for FOLR1-positive ovarian cancer.

### 3.5. Comparison with Other FRα IHC Methods

Given the critical role of FRα as a therapeutic selection biomarker, the reliability and specificity of the immunohistochemical assay used for its detection are essential.

Additional FRα-targeted antibodies and laboratory-developed tests are currently under investigation; however, their performance characteristics can vary significantly.

A recent comparative study by Deutschman et al. evaluated six non-Ventana FRα antibodies against the approved Ventana assay using ovarian cancer tissue samples [21]. The results highlighted substantial variability in staining quality and interpretability. Four of the six alternative antibodies failed to produce clean, specific membrane staining, instead exhibiting high background or nonspecific signals that compromise diagnostic accuracy [21]. Only two antibodies (from Leica Biosystems and Biocare Medical) demonstrated membrane staining patterns similar to the Ventana assay [21]. However, when these were applied using the same ≥75% tumor cell positivity threshold, both assays identified a higher number of tumors as FRα-positive compared to the companion diagnostic, effectively “overcalling” FRα expression [21].

This overestimation raises concerns about the potential for inappropriate patient selection, as individuals with tumors expressing FRα at lower levels than intended by the drug label might erroneously be deemed eligible for FRα-targeted therapies.

## 4. Tissue Selection and FRα Expression Heterogeneity

Patient selection for studies of mirvetuximab soravtansine has been based on FRα positivity of archival tumor samples assessed by IHC. In early-phase trials, including an expansion cohort study from a Phase I monotherapy study, both archival and recent biopsy specimens were used to characterize FRα expression in a heterogeneous population of relapsed ovarian cancer patients. Comparative FRα expression in matched pre- and post-treatment biopsy samples revealed high concordance of FRα expression in evaluable pre-treatment biopsies versus archival tumor samples, suggesting that archival tissue can be reliably used to identify patients with receptor-positive tumors [24].

Notably, high FRα expression, regardless of whether it was observed in archival or recent tissue, correlated with greater antitumor activity, including improved ORR, durable clinical benefit, and prolonged progression-free survival (PFS) [24]. Approximately 60% of patients in these cohorts exhibited medium to high FRα expression, with two-thirds showing high expression.

Additional studies investigating FRα expression in gynecologic tumors have revealed a wide variability across histotypes and clinical parameters. In a large cohort of 216 gynecologic malignancies, FRα positivity was observed in 45.4% of cases, with notable variation among histological subtypes: high-grade serous carcinomas (54.3%), carcinosarcomas (60%), and low-grade serous carcinomas (29.4%), while endometrioid, clear cell, mucinous, and granulosa cell tumors were negative [22].

The anatomic site of tumor sampling also influenced FRα positivity: 55.2% of primary tumor sites were positive versus 52.1% of synchronous metastatic sites and only 27.8% of recurrent/metastatic lesions. Resection specimens demonstrated a significantly higher positivity rate than biopsy specimens (54.7% vs. 25%) [22]. Interestingly, older specimens (≥19 months) showed higher FRα positivity than more recent ones (54.1% vs. 36.2%, *p* = 0.0084), likely reflecting fixation or processing differences rather than true biological changes.

A retrospective study involving 425 ovarian cancer specimens found that the site of tumor origin significantly influenced FRα positivity rates. In detail, primary ovarian and fallopian tube tumors demonstrated higher FRα expression compared to metastatic lesions. Moreover, among patients with multiple tested specimens, 37.5% had discordant FRα results, highlighting intrapatient heterogeneity and emphasizing the importance of testing the most recent and clinically relevant sample [14].

Beyond high-grade serous carcinoma, emerging evidence suggests therapeutic potential in other histologic subtypes. Rushton et al. reported strong FOLR1 positivity in 24.6% of low-grade serous ovarian cancers, especially in those lacking MAPK pathway mutations (BRAF, NRAS), suggesting a role for FRα-targeted therapies in molecularly stratified LGSOC subpopulations [25]. Saito et al. [26] also demonstrated high FRα expression in 34% of HER2-negative uterine carcinosarcomas, pointing toward further indications for mirvetuximab soravtansine in gynecologic oncology.

## 5. Conclusions

**Figure 3:**
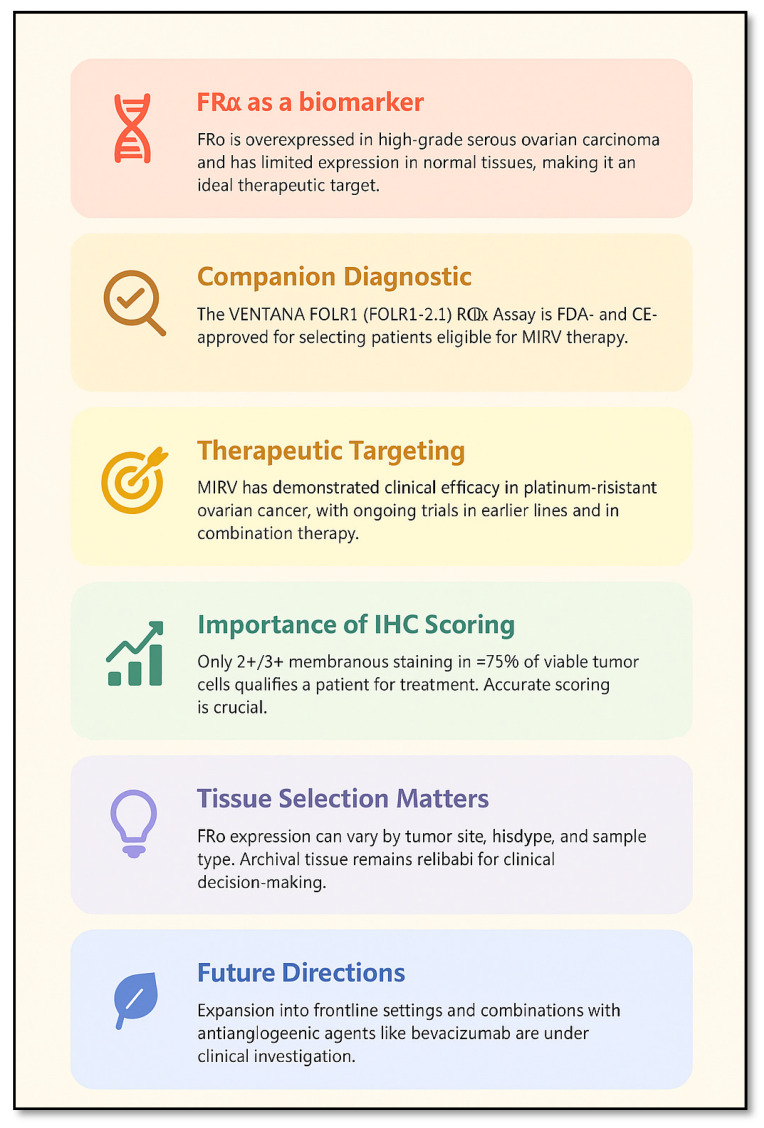
Summary of key takeaways on folate receptor alpha (FRα) in ovarian cancer (icons from BioRender).

Folate receptor alpha has emerged as a clinically relevant biomarker for the identification and selection of patients eligible for targeted therapy in ovarian cancer. The standardization of FRα testing and interpretation is essential to ensure diagnostic reproducibility and optimize therapeutic outcomes. Despite the significant clinical potential of FRα-targeted therapies, some practical challenges remain. One of the most relevant issues is the availability of suitable tumor samples for companion diagnostic testing. Inadequate fixation, small biopsies with limited viable tumor cells, or pre-analytical artifacts may render some specimens non-evaluable, particularly in recurrent or metastatic settings. Addressing these challenges through optimized tissue handling protocols and clear clinical guidelines will be essential to ensure broad and equitable access to FRα-targeted therapies. The integration of validated immunohistochemical assays into clinical workflows, combined with continued evaluation of novel FRα-targeted strategies, will be pivotal in refining patient selection and improving survival in this aggressive disease. A visual summary of the most relevant clinical, diagnostic, and therapeutic concepts related to FRα in ovarian cancer is provided in Figure 3.

## Figures and Tables

**Table 1:** Technical specifications of the VENTANA FOLR1 (FOLR1-2.1) RxDx Assay.

Feature	Description
Clone	Mouse monoclonal anti-FOLR1, clone FOLR1-2.1
Platform	BenchMark IHC/ISH
Detection Kit	OptiView DAB IHC
Cut-off for positivity	≥75% viable tumor cells with 2+/3+ membranous staining
Control tissue	Normal fallopian tube epithelium (moderate 2+ staining)
Validated specimens	FFPE primary/metastatic HGSOC tissue (resection, biopsy)
Non-validated specimens	Cytology samples, decalcified bone metastases

**Table 2:** Interpretation criteria for FRα immunohistochemical scoring in epithelial ovarian cancer.

Score	Intensity Description	Included in Scoring
0	No signal	No
1+	Faint gold/light brown, partial/circumferential	No
2+	Chocolate brown, partial/circumferential	Yes (if ≥75% cells)
3+	Thick dark brown/black, partial/circumferential	Yes (if ≥75% cells)

## Data Availability

Data are available from the corresponding author upon reasonable request.
